# *Trypanosoma brucei* (UMP synthase null mutants) are avirulent in mice, but recover virulence upon prolonged culture *in vitro* while retaining pyrimidine auxotrophy

**DOI:** 10.1111/mmi.12376

**Published:** 2013-09-09

**Authors:** Han B Ong, Natasha Sienkiewicz, Susan Wyllie, Stephen Patterson, Alan H Fairlamb

**Affiliations:** Divisional of Biological Chemistry and Drug Discovery College of Life Sciences, University of DundeeDundee, DD1 5EH, UK

## Abstract

African trypanosomes are capable of both *de novo* synthesis and salvage of pyrimidines. The last two steps in *de novo* synthesis are catalysed by UMP synthase (UMPS) – a bifunctional enzyme comprising orotate phosphoribosyl transferase (OPRT) and orotidine monophosphate decarboxylase (OMPDC). To investigate the essentiality of pyrimidine biosynthesis in *Trypanosoma brucei*, we generated a *umps* double knockout (DKO) line by gene replacement. The DKO was unable to grow in pyrimidine-depleted medium *in vitro*, unless supplemented with uracil, uridine, deoxyuridine or UMP. DKO parasites were completely resistant to 5-fluoroorotate and hypersensitive to 5-fluorouracil, consistent with loss of UMPS, but remained sensitive to pyrazofurin indicating that, unlike mammalian cells, the primary target of pyrazofurin is not OMPDC. The null mutant was unable to infect mice indicating that salvage of host pyrimidines is insufficient to support growth. However, following prolonged culture *in vitro*, parasites regained virulence in mice despite retaining pyrimidine auxotrophy. Unlike the wild-type, both pyrimidine auxotrophs secreted substantial quantities of orotate, significantly higher in the virulent DKO line. We propose that this may be responsible for the recovery of virulence in mice, due to host metabolism converting orotate to uridine, thereby bypassing the loss of UMPS in the parasite.

## Introduction

The neglected tropical disease human African trypanosomiasis (HAT) is caused by two subspecies of the protozoan parasite *Trypanosoma brucei* (*T. b. gambiense and T. b. rhodesiense*). Current drugs used for the treatment of this disease are far from ideal and are plagued by many disadvantages, such as high cost, toxic side-effects, drug resistance and lack of oral bioavailability (Fairlamb, 2003; Stuart *et al*., 2008). In order to develop suitable and more effective drugs, enzymes of essential metabolic pathways are being assessed as potential drug targets. One such pathway being examined is pyrimidine metabolism in *T. brucei*.

**Figure 1:**
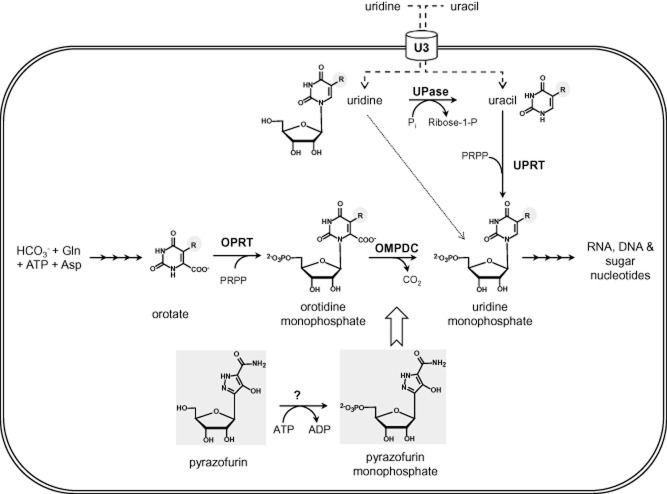
Key steps in pyrimidine *de novo* biosynthesis and salvage pathways in *T. brucei*. Orotate is converted to uridine monophosphate via the bifunctional UMP synthase (OPRT and OMPDC). Uracil is salvaged via uridine phosphorylase (UPase) and uridine via uridine phosphoribosyl transferase (UPRT). Both pyrimidines are transported via a specific pyrimidine transporter (U3). In *T. cruzi* pyrazofurin is proposed to be phosphorylated by adenosine kinase in order to inhibit OMPDC (Hammond and Gutteridge, 1983). In the structures R = H for the metabolites and R = F for the antimetabolites such as 5-fluoroorotate and 5-fluorouracil.

Pyrimidine (deoxy)-nucleotides are not only essential precursors for the synthesis of RNA and DNA, but also as CDP or UDP conjugates in the biosynthesis of glycoconjugates, complex lipids and, in some organisms, isoprenoids (via the non-mevalonate pathway), as well as in secondary metabolism in plants and in xenobiotic metabolism in animals. Most cells, including trypanosomes (Hammond and Gutteridge, 1984), can obtain their pyrimidine requirement through either *de novo* biosynthesis or salvage pathways (Fig. 1). In the *de novo* biosynthetic pathway, the precursor bicarbonate is converted into carbamoyl phosphate via carbamoyl phosphate synthase and aspartate carbamoyltransferase before cyclization to form dihydroorotate by dihydroorotase. These enzymes are fused in humans (CAD protein) whereas they are separate in trypanosomatids. Dihydroorotate is converted by dihydroorotate dehydrogenase (DHOD) to form orotic acid. The human enzyme (Enzyme Commission number 1.3.5.2), which is located in the mitochondrial membrane and transfers electrons to ubiquinone, is the target of the immunomodulatory drug, leflunomide, used to treat rheumatoid arthritis (Herrmann *et al*., 2000). In contrast, the trypanosomal enzyme (EC 1.3.98.1) is located in the cytosol and uses fumarate as an electron acceptor (Arakaki *et al*., 2008). RNAi knockdown of DHOD in *T. brucei* has been reported to retard growth *in vitro* in pyrimidine-depleted medium and DHOD-deficient trypanosomes became hypersensitive to 5-fluorouracil (Arakaki *et al*., 2008). A *umps* null has also been produced as a positive–negative selection system for genetic manipulation in *T. brucei* (Scahill *et al*., 2008). Although the normal growth phenotype observed by these authors was ascribed to sufficient pyrimidines being provided by the serum components in the standard medium, this study is open to the criticism that *Herpes simplex* virus thymidine kinase, which has broad substrate specificity for deoxypyrimidines, was used for gene replacement (Balzarini *et al*., 2002). However, virulence *in vivo* was not assessed in either of these studies. In the final steps of *de novo* biosynthesis, orotate is converted first to orotidine 5′-monophosphate (OMP) then uridine 5′-monophosphate (UMP) by the enzymes orotidine phosphoribosyl transferase (OPRT, EC 2.4.4.10) and orotidine 5′-monophosphate decarboxylase (OMPDC, EC 4.1.1.23) respectively. OPRT and OMPDC are separate monofunctional enzymes in some bacteria and certain parasites, whereas in humans they form a single bifunctional enzyme annotated as uridine monophosphate synthase (UMPS, EC 2.4.2.10). OPRT and OMPDC in *T. brucei* also comprise a single bifunctional enzyme (Tb927.5.3810). However, the order of the fusion protein is reversed (i.e. OMPDC-OPRT) and, unusually, UMPS in *T. brucei* is localized to the unique organelle, the glycosome, rather than the cytosol (Hammond and Gutteridge, 1984).

Genetic or chemically induced pyrimidine starvation results in thymineless death in prokaryotes and eukaryotes (Ahmad *et al*., 1998). These effects can be reversed by supplementation with pyrimidines which can be taken up from the growth medium via salvage pathways. To date, three pyrimidine transport systems (U1, U2 and C1) have been identified biochemically in the insect stage of *T. brucei* (Gudin *et al*., 2006). However, an additional uracil transporter (U3) has been identified in the mammalian stage that is distinct from these procyclic transporters (Ali *et al*., 2013a). This is the sole transporter expressed in bloodstream forms and U3 has high-affinity for uracil and much lower affinity for uridine and deoxyuridine (Ali *et al*., 2013a). Trypanosomes lack uridine kinase activity (Hammond and Gutteridge, 1982), so uridine is first converted to uracil via uridine phosphorylase and uracil is subsequently converted to UMP via uracil phosphoribosyltransferase. The 5-fluoropyrimidine analogues of orotate, uridine and uracil (R = F in Fig. 1) follow the same pathway (Ali *et al*., 2013a). Plasma pyrimidine levels are tightly regulated in the low micromolar range and it is thought that *de novo* biosynthesis is required to meet the high demand for pyrimidines in rapidly dividing cells. Thus, we reasoned that the non-essentiality of *de novo* synthesis *in vitro* might not apply *in vivo*.

Using a classical gene replacement with nutritional rescue strategy, we have evaluated the essentiality of *UMPS*, and thus *de novo* pyrimidine biosynthesis, in *T. brucei in vitro* and *in vivo*. Inhibitors that target pyrimidine metabolism were also tested for their antitrypanosomal activity, including pyrazofurin, a pro-drug that is metabolized to the 5′-monophosphate-derivative, which is a potent inhibitor of mammalian OMPDC (Cadman *et al*., 1978) (Fig. 1). Our findings indicate that pyrimidine biosynthesis is important for the growth of these parasites and that prolonged culture in non-physiological medium can significantly alter the virulence phenotype *in vivo* possibly by excreting orotate which is metabolized into uridine by the host.

## Results

### Generation of umps null mutants

**Figure 2:**
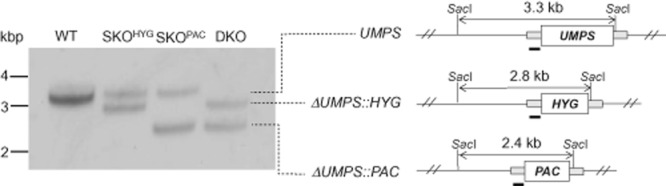
Genotypic analysis of WT, SKO and DKO cell lines. Southern blot analysis of SacI-digested genomic DNA (∼5 μg) from diploid wild-type trypanosomes (WT), Δ*UMPS*::*HYG* (SKO^HYG^); Δ*UMPS*:*PAC* (SKO^PAC^); and Δ*UMPS*::*HYG*/Δ*UMPS*::*PAC* (DKO) using a probe against the 5′-UTR of *UMPS*. A schematic representation of the *OMPDC* locus and its gene replacements is shown to the right of the blot.

The essentiality of UMPS in *T. brucei* was examined by classical sequential gene replacement, using transfection with drug-resistance genes as selectable markers. The first copy of *UMPS* was replaced with *HYG* by homologous recombination to generate a single knockout (SKO^HYG^) line resistant to hygromycin. A double knockout null mutant (Δ*UMPS*::*HYG*/Δ*UMPS*::*PAC*, referred hereafter as DKO) was then generated by replacing the second allelic copy with *PAC* in a cloned line of SKO^HYG^ resistant to both hygromycin and puromycin. The genotype of each respective stage was confirmed by Southern blot analysis of their genomic DNA (Fig. 2). SacI restriction endonuclease cuts the genomic DNA 1950-bp upstream and 13-bp downstream of the *UMPS* open reading frame yielding a ∼ 3.3 kbp fragment that can be detected by Southern analysis using a probe to the 5′-UTR. In DKO cells, this is replaced by two fragments of ∼ 2.8 kbp (*HYG*) and ∼ 2.4 kbp (*PAC*), indicating the successful integration of *HYG* and *PAC* genes and loss of *UMPS* (Fig. 2). These cells were cultured in hygromycin/puromycin-free media upon confirmation of the validity of the *umps* null mutant.

### Growth analyses of umps null bloodstream T. brucei mutants

**Figure 3:**
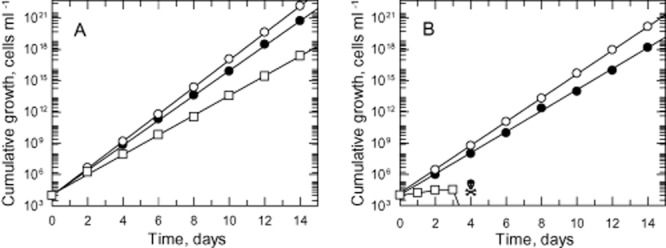
Growth analyses of WT and DKO cells *in vitro*. The cumulative growth curves after normalization for dilution at each subculture of WT (A) and DKO (B) cells were determined over two weeks in HMI9T (open circles), TBM^FCS^ (closed circles) and TBM^dFCS^ (open squares).

Our ability to generate and propagate the DKO cell line in HMI9T agrees with the previous genome-wide study by RNAi that UMPS is non-essential *in vitro* (Alsford *et al*., 2011). Thus, pyrimidines present in the normal culture medium provided by 10% fetal calf serum (FCS) and 10% Serum Plus (a proprietary supplement containing 20% FCS and other unknown components) might be serving as a metabolic bypass for the loss of *de novo* biosynthesis. Moreover, the high concentration of thymidine in HMI9T could have a sparing effect on pyrimidine requirements or theoretically thymidine could be metabolized to UMP as found in *Neurospora crassa* (Shaffer *et al*., 1975). In HMI9T medium DKO cells grew marginally slower compared with WT with doubling times of 6.2 and 5.5 h respectively (Fig. 3, Table S1). Parasites from HMI9T medium were then passaged in trypanosome basal medium plus 10% FCS (TBM^FCS^; essentially HMI9 without Serum Plus or thymidine) to determine if cells require Serum Plus for growth. In this medium, the doubling times were slightly increased to 6.1 h (WT) and 7.2 h (DKO) compared with cells grown in HMI9T (Fig. 3, Table S1). Next, the FCS in TBM^FCS^ was replaced by 10% dialysed FCS to completely eliminate pyrimidines. Under these conditions, WT cells continue to grow robustly in TBM^dFCS^ with a further increase in doubling time (7.6 h), while DKO cells were unable to grow at all and perished by day 4 (Fig. 3, Table S1). Growth of the DKO line was restored by supplementing TBM^dFCS^ with 100 μM uracil or uridine. In the presence of uracil, these parasites were able to grow at the same rate as WT cells with a doubling time of 7.4 h, whereas growth was significantly slower (doubling time of 17 h) with uridine. Supplementation with uracil or uridine only marginally enhances the growth of WT cells (Table S1). A parallel study in TBM^FCS^ and TBM^dFCS^ containing 160 μM thymidine confirmed that thymidine had no effect on growth of the WT or DKO lines (Table S1).

### Requirement for pyrimidine supplementation

**Figure 4:**
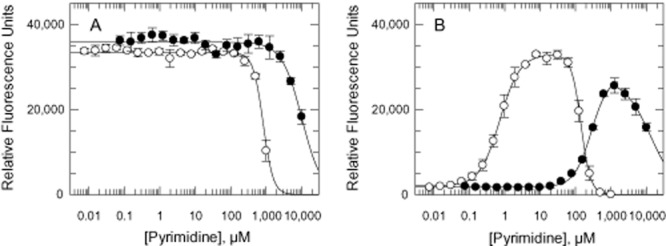
Growth of WT and DKO cells cultured in varying concentrations of uracil and uridine.A. WT cells.B. DKO cells. Symbols: plus uracil (open circles) and uridine (closed circles).

To further investigate their pyrimidine requirement, WT and DKO trypanosomes were cultured in TBM^dFCS^ supplemented with varying concentrations of pyrimidine bases (cytosine, thymine, uracil) and their corresponding nucleosides (cytidine, thymidine, uridine) (Fig. 4). Cytosine, cytidine, thymine and thymidine were unable to support growth of the DKO line at concentrations up to 1 mM. Only uracil and uridine were effective with a marked preference for uracil over uridine with GC_50_ values of 0.72 ± 0.04 μM and 250 ± 10 μM respectively (Fig. 4B). Interestingly, uracil was also found to inhibit the growth of *T. brucei* at concentrations > 100 μM. The DKO cell line was ∼ 8-fold more susceptible to inhibition compared with the WT cells with EC_50_ values of 140 ± 5 μM and 1140 ± 150 μM respectively. A similar effect was noted with uridine, where growth inhibition is observed at concentrations above 2.5 mM.

### Evaluation on UMPS as a drug target in T. brucei

A variety of inhibitors that are known to disrupt pyrimidine metabolism were examined for their effects on various *T. brucei* cell lines in TBM^FCS^. These inhibitors include pyrazofurin, 5-fluoroorotic acid, 5-fluorouracil, 5-fluorouridine, 5-bromodeoxyuridine and 6-azauridine. Pyrazofurin (as pyrazofurin monophosphate) is a potent inhibitor of OMPDC in many organisms including *Trypanosoma cruzi* (Hammond and Gutteridge, 1983), while 5-fluoroorotate is an analogue of orotate that requires metabolism by UMPS to produce 5-FUMP, which is then incorporated into RNA and sugar nucleotides (Chaudhuri *et al*., 1958; Santoso and Thornburg, 1992). Like 5-fluorouracil and 5-fluorouridine, 5-fluoroorotate is further converted to FdUMP, inhibiting thymidylate synthase as well, thereby disrupting DNA metabolism in mammalian cells (Pinedo and Peters, 1988). WT *T. brucei* cells were found to be sensitive to only three of these inhibitors: pyrazofurin, 5-fluoroorotate and 5-fluorouracil (Table 1). Surprisingly, little or no change in potency was observed when pyrazofurin was tested against DKO and SKO^HYG^ cells. In addition, sensitivity of WT cells was not affected by supplementation with uridine, uracil or orotate (Fig. S2). These results suggest that pyrazofurin is not inhibiting OMPDC in *T. brucei* and growth inhibition is possibly due to an off-target effect. In the case of 5-fluoroorotate, DKO cells were completely resistant to this inhibitor, consistent with a requirement for UMPS to convert 5-fluoroorotate into 5-FUMP. While SKO cells displayed similar sensitivity to 5-fluorouracil compared with the WT cells, the DKO parasites became ∼ 18-fold more sensitive. All cell types were completely insensitive to other uridine analogues, while sensitivity to the standard drugs eflornithine and pentamidine were unchanged. Addition of 160 μM thymidine to the medium did not affect sensitivity to pyrazofurin, 5-fluoroorotate or 5-fluorouracil.

**Table 1:** EC_50_ values of inhibitors (μM) against different cell lines cultured in TBM^FCS^

	WT	SKO^HYG^	DKO
Pyrazofurin	0.55 ± 0.02	0.42 ± 0.08	0.42 ± 0.03
5-Fluoroorotic acid	2.27 ± 0.14	1.92 ± 0.21	> 100
5-Fluorouracil	11.7 ± 0.52	8.45 ± 0.86	0.64 ± 0.08
5-Fluorouridine	> 100	> 100	> 100
5-Bromodeoxyuridine	> 100	> 100	> 100
6-Azauridine	> 100	> 100	> 100
Eflornithine	10.5 ± 0.30	7.49 ± 0.17	8.70 ± 0.26
Pentamidine	0.88 (× 10^−3^) ± 0.03	1.17 (× 10^−3^) ± 0.03	0.97 (× 10^−3^) ± 0.03

Results are means ± SEM of triplicate measurements.

### Virulence of DKO parasites in mice

**Figure 5:**
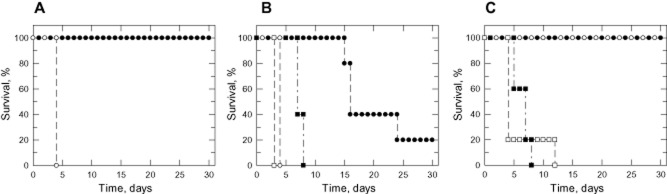
Virulence of parasites in mice. Groups of 5 mice were infected intraperitoneally with WT or DKO parasites after various culturing conditions.A. Virulence of *T. brucei* cultured in TBM^FCS^. Inoculum: 10^4^ WT parasites (open circles) or 10^4^ DKO parasites (closed circles)B. Virulence of *T. brucei* after prolonged culture in HMI9T. Inoculum: WT parasites 10^4^ (open circles) or 10^6^ (open squares); DKO parasites 10^4^ (closed circles) or 10^6^ (closed squares).C. Virulence of DKO parasites after short-term culture in TBM^FCS^ plus thymidine versus HMI9T. Inoculum: 10^4^ WT parasites cultured in HMI9T (open squares); 10^4^ DKO parasites cultured in either HMI9T (open circles) or TBM^FCS^ plus thymidine (closed circles). Another group of five mice were infected with 10^4^ DKO parasites isolated from an infected mouse in panel B (closed squares). Infections in all three studies were monitored over a period of 30 days and data are presented in the form of a Kaplan–Meier survival plot.

Mammalian blood plasma contains micromolar levels of pyrimidines, primarily uridine which seemed to be the preferred nucleoside taken up by cells (Karle *et al*., 1981; Moyer *et al*., 1985). However, *T. brucei* cannot utilize uridine efficiently, with non-physiological levels of this nucleoside required for growth (Fig. 4). With this in mind, the essentiality of pyrimidine biosynthesis for virulence of these parasites was examined in our animal model for infection. Groups of five mice were inoculated with WT or DKO parasites that had been grown in TBM^FCS^. The parasitaemia and survival of these mice were then monitored over a period of 30 days (Fig. 5A). Mice infected with WT parasites achieved a terminal parasitaemia of > 10^8^ ml^−1^ on day 4, while all five mice infected with DKO parasites remained completely free of parasites beyond 30 days. This result suggests that UMPS is essential for virulence of *T. brucei* in mice.

After subculturing in HMI9T for about 4 months, the animal studies were repeated. This was done to eliminate any possible discrepancy regarding the viability of parasites cultured in the richer HMI9T versus TBM^FCS^ that might affect their virulence. In addition, another two groups of five mice were infected with a higher dose of *T. brucei* (10^6^ cells) to investigate any inoculum-dependent effect on infectivity. Mice infected with the higher inoculum (10^6^ parasites) of WT parasites succumbed to infection earlier, on day 3 rather than day 4 (10^4^ parasites) (Fig. 5B). However, the results observed with mice infected with DKO parasites were unexpected. Mice infected with the higher dose of DKO parasites went through two waves of detectable, but not terminal, parasitaemia (i.e. > 10^5^ < 10^8^ ml^−1^) starting on day 2 before succumbing to infection on days 7 and 8. Mice infected with the lower dose showed detectable parasitaemia in three out of five mice commencing 8 days after inoculation. The three positive mice all underwent a second wave of non-terminal parasitaemia before reaching a terminal parasitaemia on days 15 and 16. A fourth mouse was found to be infected on day 20, reaching a terminal parasitaemia on day 24, while the final mouse remained parasite-free for the entire 30 days. Parasites from these mice were subsequently isolated and cultured in HMI9T medium. Analysis of these cells by Southern blotting revealed that *UMPS* was absent indicating that the infections were not due to accidental contamination by WT parasites (Fig. S1). Furthermore, these parasites remained auxotrophic for pyrimidines; their requirement for uracil or uridine supplementation as well as susceptibility to 5-fluorouracil and pyrazofurin were not changed.

Contrary to the first animal study, the second study suggests that *de novo* pyrimidine synthesis may not be essential for parasite survival *in vivo*. However, the ability of *umps* null mutants to infect mice may also be dependent on the overall health of the inoculum i.e. culture conditions prior to infection. To definitively address this possibility, a third *in vivo* study was carried out (Fig. 5C). A stabilate of the original DKO that had not been subjected to prolonged culture *in vitro* was used to establish cultures in either TBM^FCS^ or HMI9T. Groups of five mice were inoculated with 10^4^ DKO parasites cultured in either medium and infectivity compared with mice infected with WT or DKO parasites isolated from mice (DKO^M^) in the second study, both of which were cultured in HMI9T. Four out of five mice infected with WT parasites succumbed to parasitaemia on day 4 following infection, while the last mouse died on day 12. Surprisingly, the DKO^M^ parasites were highly infectious and behaved almost like WT parasites. All animals succumbed to infection between days 5 and 8, with the waves of parasitaemia observed in the second study noticeably absent. However, all mice infected with a stabilate of the DKO line that had been cultured for less than two weeks remained parasite-free beyond 30 days regardless of the medium they were cultured in. Collectively, these findings reinforced the results of our first animal study that UMPS is essential for the virulence and survival of *T. brucei in vivo*.

### Pyrimidine growth requirements

**Figure 6:**
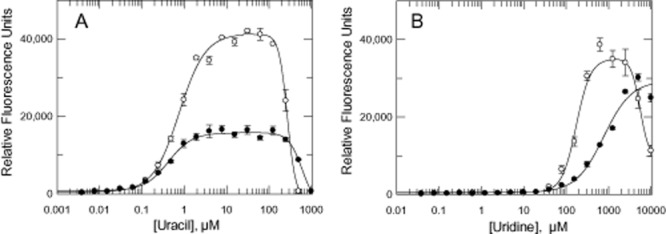
Growth of DKO and DKO^M^ cells in varying concentrations of uracil and uridine.A. Growth in varying concentrations of uracil.B. Growth in varying concentrations of uridine. Symbols: DKO (open circles) and DKO^M^ (closed circles).

To address the differences in virulence of the original DKO cell line that had not been subjected to prolonged culturing and the infectious DKO^M^, we re-examined the ability of various pyrimidines to support growth (Table 2). Uracil, uridine, UMP and deoxyuridine supported robust growth with GC_50_ values in the micromolar range. Pseudouridine, a degradation product of RNA, supported growth at concentrations above 500 μM, but we were unable to determine an accurate GC_50_ value for this pyrimidine due to its limited solubility. While cells also grew to some extent at cytidine concentrations between 1 and 5 mM, a GC_50_ value could not be determined due to toxicity above 5 mM. Total RNA from yeast (1 mg ml^−1^) was inactive. Thymidine, thymine, cytosine and dihydrouracil were unable to support growth at concentrations up to 1 mM. Although the GC_50_ values are similar between both cell types, there is a consistent minor difference when cells are grown in uracil or most other pyrimidines, where the DKO^M^ line is unable to achieve the same maximum density as the DKO line (Fig. 6A). The only exception was uridine (Fig. 6B). Differences in maximum cell density were not observed when parasites were cultured in HMI9T or TBM^FCS^ and doubling times are identical in these media provided the cells are subcultured every two days. Collectively, these results suggest there may be a subtle change in pyrimidine metabolism that has provided an advantage for DKO^M^ cells in their survival in animals but compromised growth in culture.

**Table 2:** A comparison of pyrimidine metabolism and transport in WT, DKO and DKO^M^ trypanosomes

Parameter	Units	WT	DKO	DKO^M^
Pyrimidine GC_50_				
Uracil	μM	N/A	0.81 ± 0.04 (3)	0.36 ± 0.03 (3)
Uridine	μM	N/A	190 ± 10 (2)	779 ± 70 (2)
UMP	μM	N/A	295 ± 10 (3)	320 ± 20 (3)
Deoxyuridine	μM	N/A	235 ± 10 (3)	259 ± 4 (3)
Transport kinetics				
Uracil *V*_max_	pmol s^−1^ (10^8^ cells)^−1^	0.52 ± 0.02	0.75 ± 0.06	0.64 ± 0.04
* K*_m_	μM	0.28 ± 0.04	0.51 ± 0.14	0.36 ± 0.10
Uridine *V*_max_	pmol s^−1^ (10^8^ cells)^−1^	101 ± 6	96 ± 16	100 ± 10
*K*_m_	μM	2,300 ± 500	2,400 ± 1,500	3,000 ± 1,100
Orotate production				
HMI9 medium	pmol s^−1^ (10^8^ cells)^−1^	0	5.91 ± 0.16 (3)	6.75 ± 0.14 (3)
TBM^dFCS^	pmol s^−1^ (10^8^ cells)^−1^	0	6.80 ± 0.25 (3)	8.61 ± 0.29 (3)
Orotate toxicity				
EC_50_	μM	153 ± 6 (2)	Non-toxic^a^	Non-toxic^a^

a No growth inhibition up to 1 mM.

Results are weighted means ± SEM (number of experiments in parentheses).

### Uracil and uridine transport

**Figure 7:**
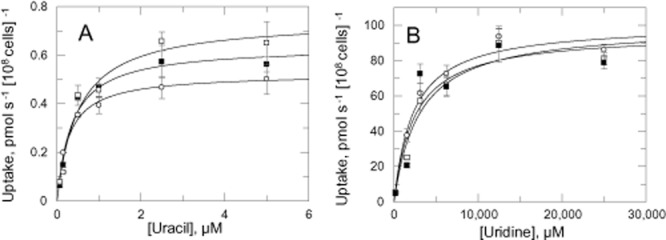
Uptake of uracil and uridine.A. Uptake of 0.07 μM ^3^H-uracil in the presence varying concentrations of unlabelled uracil.B. Uptake of 0.5 μM ^3^H-uridine in the presence of varying concentrations unlabelled uridine.Symbols: WT (open circles) and DKO^M^ (closed squares) and DKO (open squares).

The kinetics of uridine and uracil transport were investigated to determine whether upregulation in pyrimidine uptake could contribute to virulence and survival (Fig. 7). Uptake of uracil was linear for at least 2 min and obeys simple Michaelis Menten kinetics. *K*_m_ values were not significantly different, but *V*_max_ for DKO and DKO^M^ lines (Table 2) were marginally increased by ∼ 1.4 and ∼ 1.2-fold respectively. In contrast, the transport kinetics of uridine were not significantly different between the WT, DKO and DKO^M^ trypanosomes.

### Orotate production by cells

**Figure 8:**
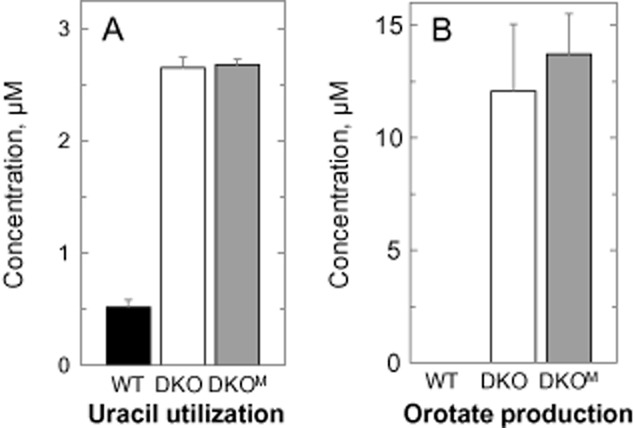
Utilization of uracil and production of orotate. Cells were cultured in HMI9T at an initial seeding density of 1 × 10^3^ cells ml^−1^, with medium harvested after 72 h and analysed by HPLC. A and B show the amount of uracil utilized and orotate produced by WT (closed bars), DKO (open bars) and DKO^M^ (gray bars) cells. The respective cell densities at this stage were 7.2 × 10^6^ cells ml^−1^ (WT) and 3.2 × 10^6^ cells ml^−1^ (DKO and DKO^M^). The initial concentration of uracil in the medium was 4.43 ± 0.26 μM.

To investigate if there were any differences in pyrimidine utilization between the DKO and DKO^M^ cells *in vitro*, spent media following 72 h of culture were analysed by HPLC. Consistent with the requirement to salvage pyrimidines for growth, both DKO cell lines took up more uracil than WT parasites ( Fig. 8A). There were no differences in cytidine, cytosine and thymidine levels in the spent medium of all three cell types. Uridine levels in the spent medium could not be accurately quantified due to the presence of 1 mM hypoxanthine which elutes very closely to uridine and interferes with the analyses.

**Figure 9:**
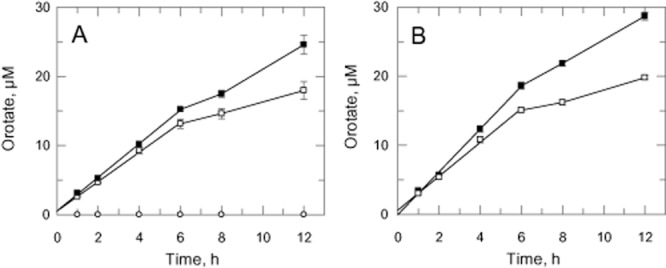
Overproduction of orotate by *umps* null mutants. Log-phase cells cultured in HMI9T were harvested, washed and resuspended in fresh medium at 1 × 10^7^ cells ml^−1^. Medium were harvested at various specified time intervals and analysed by HPLC. The production of orotate over time by DKO (open squares) and DKO^M^ (closed squares) cells were measured in HMI9T (A) and TBM^dFCS^ (B).

A striking observation from these analyses was the presence of orotic acid in the spent medium of DKO and DKO^M^, but not WT, cells (Fig. 8B). After 72 h, the concentration of orotate in the spent medium of DKO^M^ cells was noted to be consistently higher compared with the DKO parasites in these experiments. This difference was even more prominent after 96 h (at 30 ± 1.2 μM and 35 ± 1.7 μM for DKO and DKO^M^ respectively, *n* = 2, *P* = 0.07). To establish whether DKO^M^ parasites were indeed producing more orotate, log phase cells (∼ 3 × 10^6^ cells ml^−1^) cultured in HMI9T were harvested, washed and resuspended in fresh medium at 1 × 10^7^ cells ml^−1^. Media samples were collected at regular intervals and analysed for orotate production (Fig. 9A). The rate of orotate production by DKO cells in HMI9T was linear over time for the first 6 h (2.26 ± 0.07 μM h^−1^, weighted means of 3 independent experiments) and slowed thereafter reaching 19.2 ± 0.45 μM after 12 h. The initial rate of orotate production in DKO^M^ cells was ∼ 1.1-fold higher (2.39 ± 0.05 μM h^−1^) achieving a maximum ∼ 1.4-fold higher after 12 h (26.6 ± 1.31 μM). This difference in orotate production between the two cell types was more apparent when cells were incubated in a pyrimidine-free medium (TBM^dFCS^, Fig. 9B; Table 2). While the rate of orotate production by DKO cells remained unchanged (2.44 ± 0.10 μM h^−1^), the rate for the DKO^M^ cells was increased by ∼ 1.3-fold (3.12 ± 0.10 μM h^−1^, *n* = 3, *P* = 0.001). The peak amounts of orotate after 12 h were determined to be 19.6 ± 0.12 μM and 29.9 ± 0.64 μM respectively. These results suggest that DKO^M^ cells were indeed able to produce more orotate than DKO cells and are also able to further increase the rate of orotate production in a pyrimidine-free environment. Such characteristics may well confer our DKO^M^ cells with the advantage over DKO cells in relation to infection and survival in mouse host.

## Discussion

The results of our current study demonstrate that *UMPS* is essential for growth of bloodstream *T. brucei* in pyrimidine-depleted medium. Growth is supported by uracil, uridine, deoxyuridine or UMP and less robustly by pseudouridine or cytidine (UMP is presumed to be metabolized to uridine by extracellular nucleotidases or phosphatases prior to uptake). Other pyrimidines such as dihydrouracil, cytidine, cytosine, thymine or thymidine did not support growth. These pyrimidine requirements for bloodstream *T. brucei* are entirely consistent with recent transport studies that report only uracil and uridine are taken up by bloodstream forms via a single transporter, designated as U3, which is distinct from the insect stage transporters U1, U2 and C1 (Ali *et al*., 2013a). Our uracil and uridine transport kinetic parameters are in good agreement with the published values.

Loss of UMPS activity in the DKO results in several biochemical differences compared with WT trypanosomes. First, uracil transport capacity in DKO cells increases 1.4-fold and is associated with a fivefold increase in utilization of uracil from the medium to meet its nutritional requirements. Based on uracil depletion from the medium, the rate of uracil utilization can be estimated to be 0.96 pmol s^−1^ (10^8^ cells)^−1^, consistent with the transport *V*_max_ for uracil of 0.75 pmol s^−1^ (10^8^ cells)^−1^. Second, the biosynthetic intermediate, orotate, is secreted into the medium in large amounts by DKO, but not at all in WT cells. Notably, the flux through the pathway to orotate [5.9 pmol s^−1^ (10^8^ cells)^−1^] exceeds the amount of uracil estimated for growth in HMI9T by sixfold. In addition, extracellular orotate is toxic to WT, but not DKO cells (Table 2). This suggests that toxicity is due to conversion of excess orotate to UMP and other pyrimidine nucleotides. It also indicates that regulation of *de novo* synthesis must occur earlier in the pathway. The regulatory mechanism is unknown in trypanosomatids, but, in mammalian cells, this occurs via feedback inhibition by UTP and by protein phosphorylation of the first multifunctional enzyme in the pathway, CAD (Evans and Guy, 2004). Third, the complete loss of inhibitory activity of 5-fluororotate in DKO trypanosomes indicates that this molecule has to be converted into 5-FUMP by UMPS to exert its toxicity. Further conversion and incorporation into UDP-sugars and RNA, but not into deoxyuridine nucleotides or DNA, suggests the mode of action does not involve inhibition of thymidylate synthase (Ali *et al*., 2013a). This is supported by the fact that *dhfr-ts* null mutants are equally sensitive to 5-fluororotate (Sienkiewicz *et al*., 2008) and, as reported here, thymidine has no effect on growth sensitivity to fluoropyrimidines. The hypersensitivity of DKO cells to 5-fluorouracil can be explained by increased transport and salvage activity of exogenous uracil and its fluorinated analogue. Finally, pyrazofurin has been studied as a potent inhibitor of OMPDC in mammalian cells (as the monophosphate) and undergone clinical trials as an anticancer agent. In crude extracts of *T. cruzi* supplemented with ATP it potently inhibits OMPDC activity (Hammond and Gutteridge, 1983). However, the identical potency of pyrazofurin against various cell lines tested here indicates that OMPDC is not the primary target in *T. brucei* and that its anti-trypanosomal properties are due to other ‘off-target’ effects. This deserves further investigation.

So, how do these biochemical changes account for the lack of infectivity of the DKO parasites in mice? The GC_50_ and transport *K*_m_ values obtained *in vitro* suggest that uracil would be preferred over uridine *in vivo*. Radiolabelling studies in mice indicate that [5-^3^H]-uracil is rapidly catabolized with only a trace amount salvaged as nucleotides (Moyer *et al*., 1985). Unfortunately, reliable information on the plasma concentration of uracil in mice is lacking, but is ∼ 0.1 μM in humans (Coudore *et al*., 2012). If the concentration of uracil in mice is equivalent to humans, as is the case for uridine (Moyer *et al*., 1981), then the uracil level alone (GC_50_ 0.81 μM) would be insufficient to support growth. Based on our transport data the DKO cell would take up uracil from mouse plasma at 0.12 pmol s^−1^ (10^8^ cells)^−1^, considerably less than the amount estimated above for growth, i.e. 0.96 pmol s^−1^ (10^8^ cells)^−1^. The serum concentration of uridine in mouse plasma is in the range 2 to 5 μM, so uridine alone (GC_50_ 190 μM) would also be too low to support growth. At the upper plasma concentration the DKO cell would take up uridine at 0.2 pmol s^−1^ (10^8^ cells)^−1^, slightly higher than uracil. So despite the fact that the *K*_m_ and *V*_max_ for these pyrimidines differ by several orders of magnitude, rates of uptake under physiological conditions are predicted to be similar. However, it is important to note that transport of uridine at the physiological levels present in plasma will be proportional to uridine concentration since [S] < < *K*_m_. Based on current information, the contribution of deoxyuridine, UMP and other pyrimidines to growth is impossible to quantify. However, it can be concluded that the total rate of salvage of uridine plus uracil [0.32 pmol s^−1^ (10^8^ cells)^−1^] would still be about one third of the estimated amount required for normal growth.

So, what are the explanations for the surprising discovery that DKO trypanosomes regained infectivity to mice, having been maintained in long-term culture? We have carried out extensive investigations to try to answer this question. The genes of enzymes involved in pyrimidine salvage (cytidine deaminase, thymidine kinase, uracil phosphoribosyltransferase and uridine phosphorylase) were sequenced, but found to be identical in WT, DKO or DKO^M^ parasites. Upregulation of pyrimidine transport was noted in both *umps* null mutants compared with WT, but this was less in DKO^M^ than DKO, so is unlikely to account for the difference in virulence. The most significant finding was the increased production of orotate, particularly in a pyrimidine-poor environment, by the revertant (DKO^M^) cell line. By doing so, trypanosomes could possibly hijack host metabolism to convert excess orotate into uridine or other pyrimidine nucleotides. Support for this hypothesis comes from the fact that administration of orotate to rats results in a small (twofold) but sustained increase in plasma uridine and cytidine levels (Gasser *et al*., 1981; Rosenfeldt *et al*., 1998). As noted above, transport of uridine increases linearly with changes in the extracellular concentration in the physiological range, so doubling the plasma uridine level would double the rate of uptake. Whether the small, but statistically significant increase in orotate production between DKO^M^ and DKO cells is sufficient to account for the differences in virulence is open to debate, but it is clear from the foregoing discussion that subtle differences in pyrimidine levels could tip the balance between death or survival of the parasite in the mouse.

During the review process of this manuscript, a similar knockout study was published which largely agrees with our findings (Ali *et al*., 2013b). These authors also found their UMPS-deficient *T. brucei* to be infectious in their animal model. Although their model proposing upregulation in uracil transport as a compensatory mechanism to retain virulence is conceivable, it is noteworthy that our DKO^M^ cells have a marginally decreased uracil transport compared with the DKO parasites. More importantly, the authors employed *H. simplex* virus thymidine kinase (*HSVTK*) to replace the *UMPS* gene. This enzyme has broad substrate specificity for other deoxynucleosides, including deoxyuridine (Balzarini *et al*., 2002), which could have a confounding effect on pyrimidine metabolism in *T. brucei*. In addition, deletion of the second allele via loss of heterozygosity involved prolonged passage in 5-fluoroorotate and similar compensatory changes in pyrimidine metabolism to those reported here could have occurred. Notwithstanding these criticisms, both studies agree that UMPS is not a suitable drug target in the African trypanosome, in our case due to the potential for metabolic changes leading to restoration of virulence.

## Experimental procedures

### Organisms and reagents

Pyrazofurin was purchased from Berry & Associates Inc. ^3^H-uracil (36 Ci mmol^−1^) and ^3^H-uridine (20 Ci mmol^−1^) were purchased from Perkin Elmer. Pseudouridine was purchased from Carbosynth. All pyrimidines were purchased from Sigma Aldrich. Other chemicals and reagents used in this study were of the highest grade and purity available. *Trypanosoma brucei* bloodstream-form ‘single marker’ S427 (*T7RPOL TETR NEO*) was cultured at 37°C in HMI9T (Greig *et al*., 2009), supplemented with 2.5 μg ml^−1^ G418 (Geneticin, Invitrogen). Trypanosome base media (TBM) is derived from HMI11 (Hirumi and Hirumi, 1989) and consists of Iscove's modified Dulbecco's MEM supplemented with 0.05 mM bathocuproine, 1.5 mM cysteine, 1 mM hypoxanthine, 0.2 mM 2-mercaptoethanol and 1 mM sodium pyruvate. TBM was supplemented with either bovine FCS or dialysed bovine FCS (both from PAA laboratories) and other pyrimidines as detailed in the text. Cell densities for seeding cultures were determined using the CASY TT cell counter (Schärfe).

### Generation of knockout constructs

The PCR primers used to generate constructs for genetic manipulation were designed using the *T. brucei UMPS* sequence in GeneDB (Tb927.5.3810) as a template (Table S2). The accuracy of all assembled constructs was verified by sequencing. Replacement cassettes were generated by amplifying a region of DNA encompassing the 5′-untranslated region (UTR), open reading frame and 3′-UTR of *UMPS* from genomic DNA with primers 5′-UTR-NotI_s and 3′-UTR-NotI_as, using *Pfu* polymerase. This sequence was then used as a template for the amplification of the individual regions used in the assembly of replacement cassettes containing the selectable drug resistance genes puromycin *N*-acetyl transferase (*PAC*) and hygromycin phosphotransferase (*HYG*), exactly as previously described (Martin and Smith, 2005).

### Generation of T. brucei transgenic mutants

Knockout constructs were prepared using QIAprep Miniprep Plasmid Kit (Qiagen). Plasmids were linearized using NotI, precipitated with ethanol and resuspended in sterile water (1 μg ml^−1^). Wild-type trypanosomes cultured in HMI9T were electroporated using programme X-001 of the Nucleofector II electroporator (Amaxa, Cologne, Germany) (Burkard *et al*., 2007) following the addition of 5 μg of linearized DNA and reagents from the Human T cell Nucleofector kit as per manufacturers' instructions. Parasites were cloned by limiting dilution following each transfection. To generate the double knockout (DKO) of *UMPS*, the first allele was replaced with *HYG*. A single knockout cell-line (SKO^HYG^) was selected by culturing cells in the continuous presence of hygromycin (4 μg ml^−1^). Once established, the second *UMPS* allele in SKO^HYG^ cells was replaced by the *PAC* gene. The resulting DKO cell line was selected by culturing cells in the continuous presence of both hygromycin and puromycin (0.1 μg ml^−1^).

### Southern blot analysis

The 5′-UTR of *T. brucei UMPS* was amplified by PCR (using the primers previously described for generating the knockout out constructs) and the PCR DIG Probe Synthesis Kit (Roche). The resulting digoxigenin-labelled product was used as a probe. Southern analysis was carried out exactly as previously described (Wyllie *et al*., 2009) using samples of genomic DNA (5 μg) digested with the restriction endonuclease SacI.

### Concentration of pyrimidines required for half maximal growth

The ability of different pyrimidines to support the growth of the DKO lines was investigated in pyrimidine-free TBM supplemented with 10% dialysed fetal calf serum (TBM^dFCS^). The concentration of various pyrimidines required to support growth was determined in 96-well microtitre plates in a final culture volume of 200 μl per well and an initial parasite seeding density of 2.5 × 10^3^ cells ml^−1^. Stock solutions of cytidine, thymidine, uridine, pseudouridine, deoxyuridine and UMP were prepared in water, and cytosine, thymine, uracil, dihydrouracil and torula yeast total RNA (Sigma Aldrich) were dissolved in 1 mM NaOH. Appropriate amounts of solvent were included in control wells. Cultures were incubated for 72 h before cell density was determined using a resazurin-based assay (Raz *et al*., 1997; Jones *et al*., 2010). The concentration of each pyrimidine required to support 50% maximum cell growth (GC_50_) was determined by plotting cell density versus pyrimidine concentration and analysed by 4-parameter non-linear regression using GraFit.

### EC_50_ determination of inhibitors of pyrimidine metabolism

EC_50_ values for inhibitors were determined using parasites cultured in TBM plus 10% FCS (TBM^FCS^), with or without thymidine. The concentration of inhibitors to inhibit 50% growth was determined in 96-well microtitre plates in a final culture volume of 200 μl per well and an initial parasite seeding density of 2.5 × 10^3^ cells ml^−1^. All wells, including controls, contained 0.5% DMSO. EC_50_ values were determined as described above.

### In vivo studies

All animal experiments were subject to local ethical review and performed under the Animals (Scientific Procedures) Act 1986 in accordance with the European Communities Council Directive (86/609/EEC). All lines of parasites were cultured and routinely passaged in HMI9T or TBM^FCS^ plus thymidine. To avoid G418 toxicity to mice, trypanosomes were cultured in the absence of G418 for 4–12 h prior to inoculation. Groups of five mice were injected intraperitoneally with a specified number of parasites, with infections and parasitaemia monitored over a period of 30 days as previously described (Sienkiewicz *et al*., 2008). Animals reaching a lethal parasitaemia (> 10^8^ ml^−1^) were humanely killed and recorded as having died on that day.

### Pyrimidine transport

Transport of uracil and uridine were carried out as previously described (Wallace *et al*., 2002) with some modifications. *T. brucei* cells cultured in HMI9T were harvested by centrifugation (800 *g*, 10 min, 4°C), washed and resuspended (1 × 10^8^ cells ml^−1^) in transport buffer (Ali *et al*., 2013b). Transport assays were initiated by the addition of 100 μl cells to 100 μl pyrimidine in a microfuge tubes at room temperature. Uptake of ^3^H-uracil (0.07 μM) was determined in the presence of varying concentrations of unlabelled uracil (0.08 to 5 μM). At specific time intervals (30, 60, 90, 120 and 150 s) transport was terminated by centrifugation (16 000 *g*, 1 min) through silicone oil (200 μl, Medford Silicones Inc.). Microfuge tubes were flash frozen in liquid nitrogen and the bottom of the tubes containing the cell pellets were snipped off directly into scintillation vials. Pellets were solubilized in 1 M NaOH (150 μl) overnight and mixed with scintillation fluid (2 ml) before radioactivity was determined using a liquid scintillation counter. Uptake of ^3^H-uridine (0.5 μM) in the presence of varying concentrations of unlabelled uridine (195 to 25 000 μM) was similarly determined, with transport terminated at time intervals (60, 120, 300, 450 and 600 s).

### Analysis of purines and pyrimidines in media and serum

Purines and pyrimidines in samples were analysed by HPLC as previously described (Tavazzi *et al*., 2005). Analytes were separated on an ODS-2 Hypersil column (Thermo) using a Dionex UltiMate 3000 system coupled to a Dionex UltiMate 3000 variable UV detector. Samples and mobile phase were prepared as previously described (Tavazzi *et al*., 2005), with the exception that tetrabutylammonium hydroxide in the mobile phases was replaced by tetrabutylammonium phosphate (Sigma).

### Measurement of orotic acid production in medium

Log-phase cells (3 × 10^6^ cells ml^−1^) cultured in HMI9T were harvested by centrifugation (800 *g*, 10 min, 4°C), washed once in fresh HMI9T or TBM^dFCS^ and resuspended at 1 × 10^7^ cells ml^−1^). Cultures were incubated at 37°C with 5% CO_2_. At time intervals 1, 2, 4, 6, 8 and 12 h, aliquots of cultures (500 μl) were removed with medium separated from cells by centrifugation (800 *g*, 10 min, 4°C), treated and orotate levels measured by HPLC. Orotate production over time was analysed by linear regression in Grafit. Statistical analyses were carried out using Student's *t*-test with two samples equal variance. *P* < 0.05 is considered statistically significant.
