# Supplementary material for: Trypanosoma brucei (UMP synthase null mutants) are avirulent in mice, but recover virulence upon prolonged culture in vitro while retaining pyrimidine auxotrophy
Source: Mol Microbiol. 2013 Sep 9;90(2):443–55. doi: 10.1111/mmi.12376 (PMC3868941; doi:10.1111/mmi.12376)

Trypanosoma brucei (UMP synthase null mutants) are avirulent in mice, but recover virulence upon prolonged culture *in vitro* while retaining pyrimidine auxotrophy

Han B Ong, Natasha Sienkiewicz[1], Susan Wyllie, Stephen Patterson and Alan H Fairlamb

Divisional of Biological Chemistry and Drug Discovery, College of Life Sciences, University of Dundee, DD1 5EH, UK

E-mail: a.h.fairlamb@dundee.ac.uk Tel.: +44 1382 385155

Table S1 Growth of WT and DKO parasites cultured in various media

Table S2 Cloning primers

Figure S1 Genotypic analysis of WT, DKO and DKO cell lines isolated from mice

Figure S2 Effect of pyrimidines on the sensitivity of WT cells to pyrazofurin

Supporting information

Table S1 Growth of WT and DKO parasites cultured in various media. The composition of the basal medium is described in Experimental Procedures.

Trypanosome Basal Medium (TBM) Supplements	Doubling time (h)			
	WT plus thymidine	DKO plus thymidine	WT no thymidine	DKO no thymidine
10% FCS / 10% Serum Plus	5.53 ± 0.01 [a]	6.23 ± 0.01 [a]	-	-
10% FCS	6.05 ± 0.01	7.16 ± 0.02	6.11 ± 0.01	7.21 ± 0.01
10% dialysed FCS	7.63 ± 0.01	No growth	7.60 ± 0.01	No growth
10% dialysed FCS / 100 µM uracil	7.35 ± 0.03	7.42 ± 0.04	7.40 ± 0.01	7.61 ± 0.02
10% dialysed FCS / 100 µM uridine	7.35 ± 0.01	17.01 ± 0.25	7.40 ± 0.01	17.18 ± 0.10
10% dialysed FCS / 1,000 µM uridine	7.35 ± 0.02	7.22 ± 0.04	7.35 ± 0.02	7.35 ± 0.01

[a] This medium is commonly referred to as HMI9T, used for routine culture of bloodstream forms of *T. brucei* .

Table S2 Cloning primers

Upper case letters refer to nucleotides corresponding to gene sequences in *T. brucei*; lower case refers to additional sequences used in generating constructs. Restriction endonuclease sites are underlined.

Primer name	Primer sequence
5´UTR-NotI _s	5´ataagaat<u>gcggccgc</u>TACAACATGCGTTTGATCTATCAGT 3´
5´UTR-HindIII/PmeI_as	5´gtttaaacttacggaccgtc<u>aagctt</u>TTTGTAACTGTTTGCCTTTCACAAC 3´
3´UTR-PmeI/BamHI_s	5´gacggtccgtaagtttaaac<u>ggatcc</u>GTGGGTCGGAGCTCTATCTTTAAGC 3´
3´UTR-NotI_as	5´ataagtaa<u>gcggccgc</u>TACCCTTCAATGCAGAGGGCACCACAGC 3´

Figure S1. Genotypic analysis of WT, DKO and DKO cell lines isolated from mice. Southern blot analysis of SacI-digested genomic DNA (~5 µg) WT, DKO and DKO cell lines isolated from 5 different mice using a probe against the 5´-UTR of UMPS.

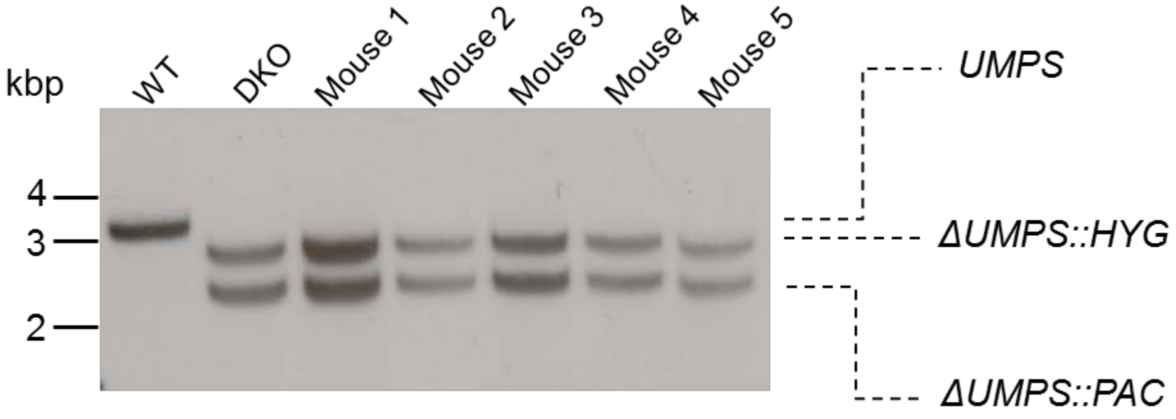

Figure S2 Effect of pyrimidines on the sensitivity of WT cells to pyrazofurin. The EC_{50} values of pyrazofurin against WT cells was determined in TBM^{dFCS} alone (closed circles; 0.42 ± 0.01 µM) or in the presence of 100 µM uracil (open squares; 0.44 ± 0.01 µM) or 100 µM uridine (closed squares; 0.51 ± 0.01 µM) or 100 µM orotate (open circles; 0.43 ± 0.02 µM). Results are the means ± S.E.M. of triplicate measurements.

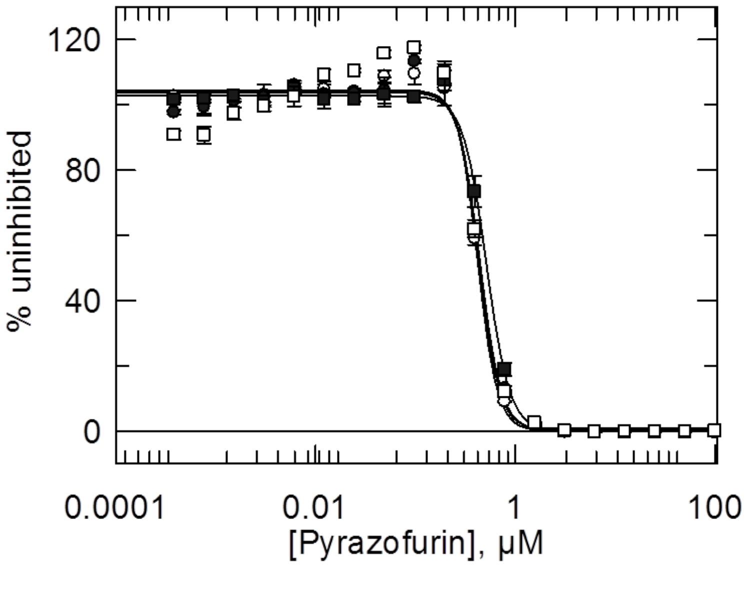

Supplement: Supplementary file 1 [file mmi0090-0443-sd1.pdf]
